# Dietary pattern and other lifestyle factors as potential contributors to hypertension prevalence in Arusha City, Tanzania: a population-based descriptive study

**DOI:** 10.1186/s12889-017-4679-8

**Published:** 2017-08-16

**Authors:** L. K. Katalambula, D. N. Meyer, T. Ngoma, J. Buza, E. Mpolya, A. H. Mtumwa, P. Petrucka

**Affiliations:** 1 School of Life Science and Bioengineering, Nelson Mandela African Institution of Science and Technology Department of Public Health, University of Dodoma, Dodoma, Tanzania; 2 School of Public Health, University of Saskatchewan, Saskatoon, SK Canada; 3 Muhimbili University of Health and Allied Sciences, Dar es Salaam, Tanzania; 4 School of Life Science and Bioengineering, Nelson Mandela African Institution of Science and Technology, Arusha, Tanzania; 5 Department of Statistics, University of Dodoma, Dodoma, Tanzania; 6 College of Nursing, University of Saskatchewan, Regina, SK Canada

**Keywords:** Hypertension prevalence, Blood pressure, Dietary pattern, Lifestyle, Arusha, Tanzania

## Abstract

**Background:**

High blood pressure is increasing worldwide, disproportionately so in developing countries. Inadequate health care systems and adoption of unhealthy lifestyles have been linked to this emergent pattern. To better understand this trend, it is imperative we measure prevalence of hypertension, and examine specific risk factors, at a local level. This study provides a cross-sectional view of urban residents of Arusha City to determine prevalence and associated risk factors.

**Methods:**

Blood pressure was measured using a digital sphygmomanometer. Interviews were conducted using the WHO STEPwise survey questionnaire to assess lifestyle factors. Dietary intake information was collected by a standardized Food Frequency Questionnaire (FFQ)**.** Descriptive statistics were used to analyze demographic characteristics. Means and standard deviations were calculated for continuous variables and percentages for categorical variables. Pearson’s Chi Square (*χ* ^2^) tests were used to determine significant risk factors for hypertension, and multivariate log binomial regression was used to reveal potential predictors of hypertension. Dietary patterns were analyzed by principal component analysis.

**Results:**

Approximately 45% of the study population was found to be hypertensive. The mean arterial blood pressure (MABP) of the sample was 102.3 mmHg (SD = 18.3). Mean systolic and diastolic blood pressure were 136.3 (SD = 30.5) and 85.3 (SD = 16.1) mmHg, respectively. Through multivariate analysis, age and body mass index were found to be independently, positively, associated with hypertension. Adherence to ‘healthy’ dietary pattern was negatively independently associated with hypertension.

**Conclusions:**

With nearly half of participants being hypertensive, this study suggests that hypertension is a significant health risk in Arusha, Tanzania. Obesity, healthy diet, and age were found to be positively associated with hypertension risk. This study did not establish any significant association between increased blood pressure and Western-dietary pattern, cigarette smoking, alcohol intake, and physical activities.

## Background

Hypertension (HTN) contributes to heart disease, kidney failure, stroke and numerous other morbidities [1]. Further, prevalence is climbing worldwide, but especially in developing countries. This is attributed to a combination of poor health systems and increasingly unhealthy lifestyles [2]. According to the World Health Organization (WHO), the highest prevalence globally is 46%, occuring in Africa [3]. Within sub-Saharan Africa, HTN prevalence varies significantly between urban and rural settings.

Hypertension can be avoided or controlled through lifestyle factors including diet, exercise, weight management, and avoiding smoking, excess alcohol and stress [4]. Because HTN does not present outward symptoms in the early stages, it often goes undetected, particularly in impoverished conditions where there is limited screening for non-communicable diseases [3]. The costs of undiagnosed and poorly managed HTN are extremely high for both the individual and society [5]. As such, it is important to understand the local conditions, allowing for better support for private and public initiatives to reduce risk, increase early detection, and improve treatment.

In this study, we conducted a cursory assessment of high blood pressure in urban Arusha City, in northern Tanzania. We established local HTN prevalence and examined specific risk factors, including dietary pattern. This study fills a knowledge gap and better prepares government, decision-makers, medical professionals, and residents to make informed policy, programming, and personal choices.

## Methods

### Study area

In keeping with economic conditions throughout East Africa, Tanzania has a very low gross domestic product [6]. Poverty rates are high (28.2% experiencing basic poverty and 9.7% living in extreme poverty); however, these rates have been improving in recent years [7]. Arusha City is located in Arusha Municipal District in northern Tanzania, close to the foot of Mount Kilimanjaro and near the border to Kenya. All residents of the district are considered urban (population 416,000) [8]. The city is divided into 25 municipal wards, four of which were randomly selected for this study.

### Participant recruitment and data collection

Eligible participants were consenting adults reporting their home address within the wards of Muriet, Sekei, Sokon I or Unga Ltd. in Arusha City. Eight interview/screening sessions, and a number of follow up sessions, took place in various public community buildings (i.e., schools, local government offices, and clinics) in each of the wards, between April and May 2016. Participants were recruited through poster advertisement to attend the sessions in exchange for free medical advice based on their screening results.

Registration was completed upon arrival on a first come, first served basis. Weight and height were recorded using a spring scale and a measuring tape affixed to the wall, respectively. Body mass index (BMI) was calculated as weight divided by height squared (kg/m^2^). Each participant’s BMI measurements were categorized as underweight, normal, overweight, and obese based on the standard cut-off points set by the WHO [9].

Blood pressure was measured at least fifteen minutes after arrival using a digital sphygmomanometer with automatic inflation (Life Brand™ BM60). Participants’ HTN status was provisionally classified based on blood pressure ranges consistent with the Seventh Joint National Committee [10]. Table 3 illustrates systolic (SBP) and diastolic blood pressure (DBP) ranges used for HTN classification. When measured SBP or DBP were in differing classes, the more severe classification was applied. Participants with high blood pressure were urged to return for re-testing the following day, or referred to an medical clinic for urgent or follow-up care, as deemed most appropriate by our medical staff. Follow-up readings were used in this analysis.

Following physical screening, participants were interviewed using the standardized Swahili translated version of the WHO STEPwise© survey questionnaire consisting of core, expanded, and optional variables [11]. The frequency of total, vigorous, and moderate intensity physical activities was calculated per WHO STEPwise© survey manual [11]. Dietary intake information was collected by a standardized Food Frequency Questionnaire (FFQ) assessing self-reported food intake over the previous year. The tool was previously developed and validated [12].

### Data analysis

The data collected were entered in Excel™ and analyzed by Statistical Analysis System™ (SAS) Version 9.4 and SPSS™ Version 21. In all, 549 subjects were included in the study. Descriptive statistics were used to analyze demographic characteristics. Means and standard deviations were calculated for continuous variables and by percentages for categorical variables. Pearson’s Chi Square (*χ* ^2^) tests were used to determine significant risk factors for HTN. Independent variables significantly associated with HTN in univariate analysis were subjected to multivariate log binomial regression model to reveal potential associations with HTN. The independent variables considered include: gender, age, education level, employment, marital status, BMI, and dietary pattern. Alpha of 0.05 was used to determine statistical significance. The final model included only the variables which were significantly associated with HTN.

Dietary patterns were analyzed by first classifying the 58 food items of the FFQ into 12 food groups. This was to minimize within-person variations in intakes of individual foods. Principal component factor analysis was conducted to derive dietary patterns based on the 12 foods/groups. Factors were rotated by an orthogonal transformation (resulting in uncorrelated factors) to achieve a simpler structure with greater interpretability. Components with an eigenvalue >1,as well as the interpretability of the factors, were considered when determining the number of factors to maintain. The factor analysis generated three major dietary patterns. The first dietary pattern was labelled ‘healthy’ as it was loaded with carbohydrate rich foods, vegetables, fruits, and spices. The second major pattern was dubbed ‘Western’ due to high meat, milk, and fat intake. The least frequent dietary pattern was characterized by high intake of sweets, roots, and tubers and legumes and was called ‘complex carbohydrate’.

## Results

### General characteristics of the sample

A total of 549 man and women from the four random wards of Arusha City participated in the screening (demographics are shown in Table 1) and all were included in this analysis. The average age was 40.7 years (SD = 12.07), with all participants between 25 and 64 years. The majority of the participants, 34.6%, were in the 35 to 44 age range, while the fewest were 55 years and older (15.8%). Females were overrepresented, constituting 57.6% (*n* = 316) of the sample. Of the 549 participants, more than two thirds (81.2%) were educated up to a primary level, 86.2% were self-employed, and more than half (64.8%) were married or cohabiting.

**Table 1:** Demographic characteristics of participants

Variable	Number	Percent (%)
Gender		
Male	233	42.4
Female	316	57.6
Age (years)		
25–34	175	31.9
35–44	190	34.6
45–54	97	17.7
55–64	87	15.8
Education level		
Up to primary level	446	81.2
Secondary level	78	14.2
College and higher levels	25	4.6
Employment status		
Employed (private and government)	60	10.9
Self-employed	473	86.2
Students	16	2.9
Marital status		
Married/cohabiting	356	64.8
Single	136	25.1
Separated/divorced	26	5.1
Widowed	27	4.9

### Blood pressure

Mean arterial blood pressure (MABP) was calculated using the formula [(2*DBP) + SBP]/3. The mean MABP of the sample was 102.3 mmHg (SD = 18.3). Means for SBP and DBP were 136.3 (SD = 30.5) and 85.3 (SD = 16.1) mmHg, respectively (averages, by gender, are shown in Table 2). Nearly 45% of the study population was classified as hypertensive, 23% being stage 1 and 21.7% being stage 2 (see Table 3).

**Table 2:** Mean arterial blood pressure values by gender

Gender	MABP	SBP	DBP
Male	102.3 (SD = 18.3)	136.3(SD = 30.5)	85.3(SD = 16.1)
Female	104.6(SD = 47.8)	138.4(SD = 63.3)	87.6(SD = 60.5)
Total	103.6 (SD = 38.2)	137.5(SD = 52)	86.6(SD = 47.1)

**Table 3:** Hypertension classification and participant prevalence based on systolic and diastolic blood pressure readings

	Systolic Blood Pressure range (mmHg)	Diastolic Blood Pressure Range (mmHg)	Distribution of participants
Normotensive	<120	<80	125 (22.7%)
Pre-hypertensive	120–139	80–89	179 (32.6)
Hypertensive – Stage 1	140–159	90–99	126 (23.0)
Hypertensive – Stage 2	≥160	≥100	119 (21.7)

Blood pressure range criteria from the Seventh Joint National Committee [10]

## Univariate analysis of potential contributing factors of hypertension in Arusha, Tanzania

The results of univariate analysis demonstrate potential risk factors for HTN, shown in Table 4. The overall prevalence of HTN (Stage 1 and 2) was 44.7% and pre-hypertension is a further 32.6%. Males were more likely to be hypertensive than females (47.6% versus 42.4%) though the difference was not statistically significant (*p* = 0.22). Females were more likely to be pre-hypertensive (34.8% versus 29.4%). Hypertension risk was not significantly associated with the ‘Western’ diet or ‘complex carbohydrate’ diet (*p* = 0.518 and *p* = 0.352, respectively). Alcohol drinking at least once in the previous year (*p* = 0.083), and current smoking (*p* = 0.341) were also not significantly associated with HTN risk. As anticipated, ‘healthy’ dietary pattern was negatively associated with HTN (*p* = 0.01). Hypertension risk increased with age (*p* = 0.0001) and BMI (*p* = 0.005), but decreased with higher levels of education (*p* = 0.042) and doing vigorous physical activities (though not significantly, *p* = 0.058). The prevalence of HTN increased from 26.3% in the youngest age group to 70.1% in the oldest (p= > 0.0001). Risk doubled when comparing underweight individuals with obese (*p* = 0.005). The risk of HTN increased among those who did not meet criteria for moderate or total physical activities although the difference was not statistically significant (*p* = 0.103 and *p* = 0.167 respectively). There was statistically significant difference in HTN between different employment statuses where the self-employed individuals were more likely to be hypertensive (46.7%) than students or employees (*p* = 0.024).

**Table 4:** Univariate analysis of demographic variables among hypertensive participants (Stage 1 and 2)

Variable	Hypertensive (Stage 1 and 2) n (%)	*P* value
Gender		
Male	111 (47.6)	0.22
Female	134 (42.4)	
Age (Years)		
18–34	46 (26.3)	0.000
35–44	82 (43.2)	
45–54	56 (57.7)	
55–79	61 (70.1)	
Education level		
Up to primary level	45 (57.7)	0.042
Secondary level	190 (42.6)	
College and higher levels	10 (40)	
Employment status		
Employed (private and government)	21 (35)	0.024
Self employed	221 (46.7)	
Students	3 (18.8)	
Marital status		
Married/cohabiting	162 (45.5)	0.057
Single	51 (37.5)	
Separated/divorced	16 (61.5)	
Widowed	16 (59.3)	
BMI status		
Underweight	5 (23.8)	0.005
Normal	94 (38.5)	
Overweight	78 (50.0)	
Obese	68 (53.1)	
Healthy Dietary Pattern		
Yes	184 (42)	0.01
No	61 (55)	
Western Dietary Pattern		
Yes	177 (44.7)	0.518
No	68 (44.4)	
Complex Carbohydrate Dietary Pattern		
Yes	195 (44.1)	0.352
No	50 (46.7)	
Vigorous physical activities		
Met criteria	39 (36.4)	0.058
Didn’t meet criteria	206 (46.6)	
Moderate physical activities		
Met criteria	133 (41.7)	0.103
Didn’t meet criteria	112 (48.7)	
Total physical activities		
Met criteria	143 (42.3)	0.167
Didn’t meet criteria	102 (48.3)	
Alcohol intake last year		
Yes	127 (48.5)	0.083
No	118 (41.1)	
Current smokers		
Yes	34 (50)	0.341
No	211 (43.9)	

## Multivariate analysis of potential contributors to hypertension in Arusha Tanzania

Univariate analysis showed that age, education level, employment, BMI, and ‘healthy’ dietary pattern were significantly associated with HTN. These variables were subjected to multivariate logistics binomial regression. Upon building the model, education level, vigorous physical activity and employment were no longer significant correlates and were removed from the final model. Age, BMI and ‘healthy’ dietary pattern were independently associated with HTN (Table 5) and were maintained.

**Table 5:** Results of multiple log binomial models for significant predictors of hypertension

Variable	Parameter Estimates	Standard Error	*P*-Value	ARR	95% CI of ARR
Age (Years)					
25–34	Reference				
35–44	0.4948	0.1501	0.001	1.64	1.22–2.20
45–54	0.8125	0.1518	<0.0001	2.25	1.67–3.03
55–64	0.9147	0.1458	<0.0001	2.50	1.88–3.32
BMI(kg/m^2^)					
< 18	−0.8276	0.3905	0.0341	0.44	0.20–0.94
18–24.9	−0.2427	0.104	0.0196	0.78	0.64–0.96
25–29.9	−0.0675	0.1009	0.5032	0.93	0.77–1.14
30+	Reference				
Healthy dietary pattern					
Yes	−0.1886	0.0957	0.0488	0.82	0.68–0.99
No	Reference				

Ultimately, a positive association between HTN and age was supported. The risk of being hypertensive for groups aged 35–44 (ARR = 1.64, CI: 1.22–2.20) and 45–54 (ARR = 2.25, CI: 1.67–3.03) was almost twice that of the 18-34 year olds, and three times greater for those aged 55–79 (ARR = 2.50, CI: 1.88–3.32). Compared to obese participants, those who were underweight (ARR = 0.44, CI: 0.20–0.94) and normal weight (ARR = 0.78, CI: 0.64–0.96) had significantly lower prevalence of HTN. Though not significant, overweight individuals are also less likely to be hypertensive than the obese (ARR = 0.93, CI: 0.77–1.14). Following a ‘healthy’ dietary pattern was significantly negatively correlated with HTN (ARR = 0.82, CI: 0.68–0.99).

## Discussion

There are few comparable studies of HTN prevalence and associated risk factors specific to the Arusha City, Tanzania. Our co-authors (JB and PP) have previously investigated HTN prevalence in a comparative study amongst urban Arusha Massai (27.7%) and their rural, traditional counterparts (10.7%) [13]. In another study of predominantly Maasai participants migrating from nearby Simanjiro to Arusha City, 21.4% HTN prevalence was described [14]. Maasai in Arusha City are a minority population, and tend to have very different body morphology and dietary practices than the other, more predominant, residents of Arusha City. Maasai tend to grow very tall, are typically very thin, and traditionally live a nomadic life, not cultivating or relying heavily on carbohydrate rich foods, instead deriving the majority of nutrition from meat and dairy from their cattle and goats. It would not be appropriate to compare findings from studies of Maasai to our study population as diet and body type are known correlates to HTN risk. Further, the Simanjiro study specifically excluded participants with an established HTN diagnosis and therefore does not represent the overall HTN prevalence [14].

Two older studies (1999 and 2002), found HTN rates in Tanzanian’s largest coastal city, Dar es Salaam, to be similarly around 30% [15, 16]. Given the worldwide trend towards increasing HTN predominance, those figures are likely an underestimation of current rates [2]. In the city of Mwanza, in northwestern Tanzania, HTN amongst males only was 23.7% [17]. We would anticipate a typical diet in Mwanza to be derived of a greater proportion of fish as it is located on Lake Victoria, where fishing is a major industry. This may account, partially, for HTN risk difference. In a recent study across four countries in sub-Saharan Africa, age standardized HTN prevalence was 25.9% [18]. On the other hand, researchers have found much higher HTN rates in other parts of Africa, including in Cameroon, where 47.5% of the study population was found to be hypertensive [19].

Our findings demonstrated a higher-than-typical HTN prevalence in our Arusha City study than suggested by the aforementioned Maasai or nearby studies. In part, this may be attributed to our methodology and limited resources which did not allow for repeat blood pressure analysis per recommended diagnostic guidelines [10]. Anxiety with the screening process and unfamiliarity with blood pressure equipment can elevate initial blood pressure readings [14]. Overall, our results were consistent with the extant literature with respect to major risk factors for HTN including male gender, increased BMI [13–15, 17, 19, 20], and age [14, 15, 17, 19].

Our study demonstrated the protective effect of eating a healthy diet, but did not establish a significant risk between unhealthy ‘Western’ diets and increased HTN. Njelekela et al. have shown that salt intake and coconut milk consumption are significantly correlated with increased blood pressure in men and women, respectively, in a study of Tanzanian 47–57 year olds [21]. They also demonstrated that, whether one lives pastorally, rurally or in an urban space, there are marked differences in the overall foods sources most drawn from, which, naturally has an effect on HTN risk. This is consistent with the migration study by Unwin et al., who found a protective effect of consuming local porridge, and showed a marked change in eating habits and increase weight and waist circumference when moving from rural to an urban environment [14].

## Limitations

There are limitations to our study that should not be overlooked when interpreting these findings. The methodology used to establish HTN risk is not-conclusive. As blood pressure does not remain consistent, and can be made to temporarily elevate, averages of multiple blood pressure readings over time are customary [10]. Further, volunteer bias likely favored participation by unemployed or underemployed individuals who could wait most of the day for a free medical consultation. This same population may suffer lower socioeconomic status and lower health status than the average resident in Arusha City, which may play a role in HTN risk. Our study size of 549 participants represents a minute portion of the Arusha City population.

With respect to dietary pattern, salt was not specifically addressed in the FFQ. Doing so may have offered an additional depth of understanding as dietary salt is known to be associated with blood pressure. As with all principal component factor analysis, determining the number and labels of dietary patterns, and interpretation of results, is somewhat subjective [22]. Further, this study does not consider the confounding effect of additional HTN risk factors such as stress.

## Conclusion

This study confirms the high prevalence of HTN in developing countries as it has been reported in other studies. Evidence from this study demonstrates that diet, obesity and unhealthy lifestyles have a role to play in the trend of rising HTN levels in developing countries. Hypertension, if not controlled, will result into other severe forms of cardiovascular diseases. In the worldwide crisis of cardiovascular disease, it is important to understand local contributing factors and conditions. This study has better clarified how eating patterns in Arusha are related to HTN and has established an estimate of HTN prevalence in four local wards. Taken together, these findings can be used to encourage policy-makers to place HTN screening, literacy and prevention campaigns at the forefront of their priorities.

## Abbreviations

BMI: Body mass index
CI: Confidence Interval
DBP: Diastolic Blood Pressure
FFQ: Food Frequency Questionnaire
HTN: Hypertension
SBP: Systolic Blood Pressure
WHO: World Health Organization
